# SIMPLE: implementation of recommendations from international evidence-based guidelines on caesarean sections in the Netherlands. Protocol for a controlled before and after study

**DOI:** 10.1186/1748-5908-8-3

**Published:** 2013-01-03

**Authors:** Sonja Melman, Ellen NC Schoorel, Carmen Dirksen, Anneke Kwee, Luc Smits, Froukje de Boer, Madelaine Jonkers, Mallory D Woiski, Ben Willem J Mol, Johannes PR Doornbos, Harry Visser, Anjoke JM Huisjes, Martina M Porath, Friso MC Delemarre, Simone MI Kuppens, Robert Aardenburg, Ivo MA Van Dooren, Francis PJM Vrouenraets, Frans TH Lim, Gunilla Kleiverda, Paulien CM van der Salm, Karin de Boer, Marko J Sikkema, Jan G Nijhuis, Rosella PMG Hermens, Hubertina CJ Scheepers

**Affiliations:** 1 GROW- School for Oncology and Developmental Biology, Department of Obstetrics and Gynaecology, Maastricht University Medical Centre, P.O. Box 5800, Maastricht, AZ, 6202, The Netherlands; 2 Department of Clinical Epidemiology and Medical Technology Assessment (KEMTA), Maastricht University Medical Centre, P.O. Box 5800, Maastricht, AZ, 6202, The Netherlands; 3 Department of Obstetrics and Gynaecology, University Medical Hospital Utrecht, P.O. Box 85090, Utrecht, AB, 3508, The Netherlands; 4 Department of Epidemiology, Caphri School for Public Health and Primary Care, Maastricht University Medical Centre, Maastricht, The Netherlands; 5 Department of Obstetrics and Gynaecology, University Medical Centre Groningen, P.O. Box 30001, Groningen, RB, 9700, The Netherlands; 6 Department of Obstetrics and Gynaecology, Ruwaard van Putten Hospital, P.O. Box 777, Spijkenisse, GA, 3200, The Netherlands; 7 Department of Obstetrics and Gynaecology, Radboud University Nijmegen, P.O. Box 9101, Nijmegen, HB, 6500, The Netherlands; 8 Department of Obstetrics and Gynaecology, Academic Medical Centre, University of Amsterdam, P.O. Box 22660, Amsterdam, DD, 1100, The Netherlands; 9 Department of Obstetrics and Gynaecology, Zaans Medical Centre, P.O. Box 210, Zaandam, EE, 1500, The Netherlands; 10 Department of Obstetrics and Gynaecology, Tergooi Hospital Blaricum, P.O. Box 10016, Hilversum, DA, 1201, The Netherlands; 11 Department of Obstetrics and Gynaecology, Gelre Hospital, P.O. Box 9014, Apeldoorn, DS, 7300, The Netherlands; 12 Department of Obstetrics and Gynaecology, Maxima Medical Centre, P.O. Box 7777, Veldhoven, MB, 5500, The Netherlands; 13 Department of Obstetrics and Gynaecology, Elkerliek Hospital, P.O. Box 98, Helmond, AB, 5700, The Netherlands; 14 Department of Obstetrics and Gynaecology, Catharina Hospital, P.O. Box 1350, Eindhoven, ZA, 5602, The Netherlands; 15 Department of Obstetrics and Gynaecology, Orbis Medical Centre, P.O. Box 5500, Sittard, MB, 6130, The Netherlands; 16 Department of Obstetrics and Gynaecology, St. Jans Hospital Weert, P.O. Box 29, Weert, AA, 6000, The Netherlands; 17 Department of Obstetrics and Gynaecology, Atrium Medical Centre Parkstad, P.O. Box 4446, Heerlen, CX, 6401, The Netherlands; 18 Department of Obstetrics and Gynaecology, Ijsselland Hospital, P.O. Box 690, Capelle a/d Ijssel, AR, 2900, The Netherlands; 19 Department of Obstetrics and Gynaecology, Flevohospital, P.O. Box 3005, Almere, EG, 1300, The Netherlands; 20 Department of Obstetrics and Gynaecology, Meander Medical Centre, P.O. Box 1502, Amersfoort, BM, 3800, The Netherlands; 21 Department of Obstetrics and Gynaecology, Hospital Rijnstate, P.O. Box 9555, Arnhem, TA, 6800, The Netherlands; 22 Department of Obstetrics and Gynaecology, ZG Twente, P.O. Box 7600, Almelo, SZ, 7600, The Netherlands; 23 Scientific Institute for Quality of Healthcare (IQ healthcare), Radboud University Nijmegen Medical Centre, P.O. Box 9101, Nijmegen, HB, 6500, The Netherlands

## Abstract

**Background:**

Caesarean section (CS) rates are rising worldwide. In the Netherlands, the most significant rise is observed in healthy women with a singleton in vertex position between 37 and 42 weeks gestation, whereas it is doubtful whether an improved outcome for the mother or her child was obtained. It can be hypothesized that evidence-based guidelines on CS are not implemented sufficiently.

Therefore, the present study has the following objectives: to develop quality indicators on the decision to perform a CS based on key recommendations from national and international guidelines; to use the quality indicators in order to gain insight into actual adherence of Dutch gynaecologists to guideline recommendations on the performance of a CS; to explore barriers and facilitators that have a direct effect on guideline application regarding CS; and to develop, execute, and evaluate a strategy in order to reduce the CS incidence for a similar neonatal outcome (based on the information gathered in the second and third objectives).

**Methods:**

An independent expert panel of Dutch gynaecologists and midwives will develop a set of quality indicators on the decision to perform a CS. These indicators will be used to measure current care in 20 hospitals with a population of 1,000 women who delivered by CS, and a random selection of 1,000 women who delivered vaginally in the same period. Furthermore, by interviewing healthcare professionals and patients, the barriers and facilitators that may influence the decision to perform a CS will be measured. Based on the results, a tailor-made implementation strategy will be developed and tested in a controlled before-and-after study in 12 hospitals (six intervention, six control hospitals) with regard to effectiveness, experiences, and costs.

**Discussion:**

This study will offer insight into the current CS care and into the hindering and facilitating factors influencing obstetrical policy on CS. Furthermore, it will allow definition of patient categories or situations in which a tailor-made implementation strategy will most likely be meaningful and cost effective, without negatively affecting the outcome for mother and child.

**Trial registration:**

http://www.clinicaltrials.gov: NCT01261676

## Background

The worldwide rise in caesarean section (CS) rate is a major healthcare issue, with rates reported as high as 32% in the United States (US) and 37% in Brazil [1,2]. In the Netherlands, the overall CS rate has increased from 8.1% to 13.6% over the recent decade. Although this rise is relatively low compared to other countries, a striking detail is that the most impressive rise, in absolute numbers, was among healthy women with a singleton in vertex position between 37 and 42 weeks gestation [3]. However, an increasing CS rate does not imply an improved outcome for mother and infant [4]. CS are associated with an increased risk of maternal mortality as well as serious morbidity, such as admission to the intensive care unit (Odds Ratios (ORs) between 30.8 and 63.4), hysterectomy (ORs between 3.2 and 13.5), and puerperal infection (OR 3.0) [5-7].

Besides the short-term risks, CS have an impact on the mother’s future reproductive health, for example uterine rupture, placenta praevia, or placenta accreta [8,9].

There is no evidence suggesting a better neonatal outcome from the increased CS rate in terms of mortality, intracranial haemorrhage, or impaired neurological development in the general population [10,11]. In fact, an elective CS performed before 39 completed weeks is associated with respiratory distress and admission to the neonatal intensive care unit [11,12].

The question arises what causes the worldwide increase in CS rate considering the fact that in most situations there are no apparent benefits of a CS for mother and child; the costs are higher compared to vaginal birth [5]; and the incidence of both maternal and neonatal complications are increased. There are concerns about the increasing rate of planned CS as well as a declining rate of vaginal birth after a previous CS (VBAC) in the US and Australia [13,14].

To optimize CS practice, the Royal College of Obstetricians and Gynaecologists (RCOG) developed an evidence-based guideline (NICE: National Institute of Clinical Evidence) with clear recommendations for obstetric care. Similar recommendations, which have a direct effect on the decision to perform a CS, are also mentioned in the different guidelines of the Dutch Society of Obstetrics and Gynaecology (NVOG), Society of Obstetricians and Gynaecologists of Canada (SOGC), American College of Obstetrics and Gynaecology (ACOG), and National Guideline Clearinghouse from the US department of health and human services (NGC).

Despite the introduction of evidence-based guidelines, the CS rate continues to increase. We hypothesize that poor adherence to the guidelines plays a key role in the rising CS rate. In order to optimize adherence to the CS guidelines, the stepwise model by Grol can be used to select the proper strategies [15,16]. The first step in this model is to analyze the current care (measured by valid quality indicators) compared to the optimal care as described in evidence-based guidelines, and to determine which barriers and facilitators might influence the implementation of optimal care. Subsequently, a tailor-made implementation strategy can be developed with activities applied to the determined barriers. In the last step, the strategy is executed and evaluated in terms of effectiveness, feasibility, and costs*.*

In view of the rising CS rate, this study aims are:

1. To develop a set of quality indicators on the decision to perform a CS based on key recommendations of both Dutch and international guidelines.

2. To gain insight into actual adherence of Dutch gynaecologists to guideline recommendations on the performance of CS.

3. To explore barriers and facilitators that have a direct effect on application of guideline recommendations regarding CS.

4. To develop, execute and evaluate a strategy in order to improve care and possibly decrease the CS incidence for a similar neonatal outcome, based on the information gathered in steps two and three.

## Methods

The four aims were approached in four parts: the development of quality indicators, the assessment of current care, the identification of barriers and facilitators, and the development of a tailored implementation strategy and executing and evaluating this strategy in a clustered controlled before-and-after study (CBA).

### The development of quality indicators

#### Design and methods

In order to measure current Dutch practice on CS, quality indicators regarding the process, structure, and outcome of care need to be developed. This will be achieved according to the RAND-modified Delphi method [17,18]. The indicators will be based on key recommendations extracted from the guidelines of several international obstetric organisations (RCOG, NVOG, SOGC, ACOG and NGC*)*. These key recommendations will be evaluated in two rounds by an independent expert panel consisting of Dutch obstetricians and midwives. In the first round, a questionnaire will be developed on three subjects (planned CS, emergency CS, and methods to reduce the CS rate). The questionnaire will be sent to the experts who will be asked to individually rate the key recommendations on a 9-point Likert scale ranging from 1 to 9 (‘not relevant’ to ‘extremely relevant’ for measuring the quality of CS care). Furthermore, a ranking of the key recommendations will be asked per subject to ultimately extract those indicators considered to be most important for quality-of-care measurement. The experts also have the opportunity to add comments or suggest additional recommendations they consider suitable as a quality indicator. The returned questionnaires will be analysed based on the ratings of the recommendations on the 9-point Likert scale, and the median score of these items will be calculated and rated as described previously by Campbell [19]. Furthermore, scoring variables reflecting the ranking of the items in each of the three subjects will be developed (*e.g.*, in a top 3 ranking, a first ranking creates 3 points, a second ranking 2 points, and a third ranking 1 point).

The second round consists of a consensus meeting where the experts will receive their individual as well as the overall results of the first round to promote discussion. The aim of this meeting is to reach consensus on those recommendations that are most suitable for assessing the quality of care on performing CS. After consensus is reached, the recommendations will be operationalized into a set of measurable quality indicators.

### Study population and setting

A representative, national expert panel consisting of obstetricians and midwives (about 12 to 15 experts) will be invited. The obstetricians and midwives will have worked at various types of hospitals, ranging from small regional hospitals to university hospitals.

### Outcome measures

The outcome of the first step of the study is a set of valid quality indicators regarding the decision to perform a CS which can then be used to measure the current practice.

### The assessment of current care

#### Design and methods

A retrospective medical record search based on the set of quality indicators will be performed in order to assess the Dutch gynaecologists’ adherence to the CS guideline recommendations. Adherence to these indicators will be quantified, as well as the variation in care and adherence between the participating hospitals. To gain insight into the current Dutch care compared to international care, the CS percentages of the different risk groups will be calculated according to the Robson classification (Table 1) [20]. Furthermore, the maternal mortality or severe acute morbidity (Table 2) [21], and perinatal mortality or serious morbidity (pH <7.00, Apgar 5 min <7, and NICU admission) will be noted. This study will provide us with information about current practice on CS in the Netherlands and insight into the effects on outcome of mother and child.

**Table 1:** Ten-group classification according to Robson

**Groups:**	
1.	Nulliparous, single cephalic, >37 weeks, in spontaneous labour
2.	Nulliparous, single cephalic, >37 weeks, induced or CS before labour
3.	Multiparous (excluding previous CS), single cephalic, >37 weeks, in spontaneous labour
4.	Multiparous (excluding previous CS), single cephalic, >37 weeks, induced or CS before labour
5.	Previous CS, single cephalic, >37 weeks
6.	All nulliparous breeches
7.	All multiparous breeches (including previous CS)
8.	All multiple pregnancies (including previous CS)
9.	All abnormal lies (including previous CS)
10.	All single cephalic, < 36 weeks, (including previous CS)

**Table 2:** Inclusion criteria for severe acute maternal morbidity

**Group 1: ICU admission**	
-	Admission to ICU or coronary care unit, other than for standard postoperative recovery
**Group 2: Uterine rupture**	
-	Clinical symptoms (pain, fetal distress, acute loss of contractions and haemorrhage) that led to an emergency CS, at which the presumed diagnosis of uterine rupture was confirmed
-	Peripartum hysterectomy or laparotomy for uterine rupture
**Group 3: Eclampsia/ HELLP syndrome**	
-	Eclampsia
-	HELLP syndrome only when accompanied by liver haematoma or rupture
**Group 4: Major obstetric haemorrhage**	
-	Transfusion need of > 4 units of packed cells
-	Embolisation or hysterectomy for major obstetric haemorrhage
**Group 5: Miscellaneous**	
-	Other cases of severe maternal morbidity to the opinion of the treating obstetrician, not to be included in group 1-4

#### Study population and setting

In order to create a representative view of the current obstetrical care in The Netherlands, a multi-centre study will be carried out. Twenty hospitals of different Dutch regions will participate in this study, including university teaching hospitals, non-university teaching hospitals and non-university, non-teaching hospitals. The present study will take place in the setting of a Dutch Obstetric Research Consortium in which all the participating hospitals collaborate.

In the participating hospitals, data on basic obstetrical care and adherence to the quality indicators will be collected. Per hospital 100 women will be selected from the local database: 50 women who delivered by CS and a random set of 50 women who delivered vaginally in the same time period. Exclusion criteria will be a major fetal congenital malformality and fetal death prior to onset of delivery.

#### Outcome measures

The main outcome is adherence to the guideline recommendations, based on the adherence to the quality indicators. Therefore, basic obstetrical data and indicator specific data will be gathered. For example, consider the indicator ‘every woman with a child in breech presentation at 34 to 36 weeks gestation should be offered external cephalic version unless a contraindication for external cephalic version is present.’ This implies that we need to assess the incidence of breech presentation at 34 to 36 weeks gestation, as well as data that show whether an external cephalic version is being offered. Furthermore, we will note in which cases this procedure was not offered for a valid reason. This will allow us to determine the frequencies of adherence for this indicator.

The secondary outcomes are the number of preventable CS, and Dutch practice as compared to international data using the Robson criteria.

#### Sample size considerations

Assuming an adherence to the guidelines of 75%, an alpha of 0.05, and a precision of the estimation of 5%, 300 patients must be included. However, this number has to be adapted to take clustering of data across clinicians and within obstetrical departments into account. Assuming an intra-cluster correlation (ICC) of 0.2 and 80 professionals in 20 hospitals, 960 medical records need to be analysed. In order to compensate for loss to follow-up or incomplete data, 1,000 women with a CS will be included in 20 hospitals within a timescale of three to four months. In order to enable the calculation of specific events, as described in ‘outcome measures,’ a random selection of 1,000 women with a vaginal birth will be included. Thus, there will be 2,000 participants, *i.e.*, 1,000 women after a caesarean delivery and 1,000 women after a vaginal birth. Sampling fraction will be adjusted to the fraction of women with CS in each individual hospital.

#### Data analysis

The frequencies of adherence per quality indicator will be calculated. This will be calculated by dividing the total number of women who apply for an indicator and for whom care was appropriate by the total number of women who apply for an indicator. For example, women with a child in breech presentation between 34 to 36 weeks of gestation, without contraindication for external cephalic version, should have been offered an external cephalic version. Adherence is the total number of women with a child in breech presentation between 34 to 36 weeks of gestation without contraindication for external cephalic version, in whom an external version was offered, divided by the total number of women with a child in breech presentation between 34 to 36 weeks of gestation without contraindication for external cephalic version.

### Barrier and facilitator study

#### Design and methods

To determine the barriers and facilitators that influence the decision to perform a CS for healthcare professionals and patients, a qualitative study will be performed. The setting for guideline implementation will be analysed. Focus group interviews will be held among healthcare professionals (obstetricians, residents, and midwives) to discover factors that determine the decision to perform a CS or not. The interviewer will explore the following categories of influencing factors: features of the guidelines itself; features of the target group of professionals who should use the guidelines; features of patients who have to accept or contribute to the use of the guidelines; features of the social setting and social network of the professionals; and features of the organizational, economic, and administrative context. Remarks by professionals will be classified into categories of potential determining factors following this theoretical framework. The ‘prevalence’ of the features mentioned in the focus group interviews will be quantified in a survey with questionnaires among the different professionals.

Similarly, depending on the outcome of the current care study, semi-structured interviews will be held with patients in a detailed study to discover relevant factors that influence the patients’ decision to choose a CS or vaginal delivery.

#### Study population and setting

In different hospital types (university, non-university teaching, and non-university non-teaching hospitals) interviews will be held among healthcare professionals (obstetricians, residents and midwives) as well as patients. Focus group interviews among 8 to 12 healthcare professionals will be planned. To assess whether the factors mentioned in the focus group interviews are structural, the ’prevalence’ of these factors will be assessed using a survey with questionnaires among obstetric gynaecologists, residents, and midwives in the Netherlands. The questionnaires will be sent to the professionals via email addresses we will obtain from the national professional organisations of both professions. Among patients, semi-structured interviews will be held. These patients will be selected in the abovementioned hospitals from the current care study. The interviews will be conducted with those women belonging to the non-adherence subgroups (such as breech, non-progressing labour, or maternal request) to whom a possible implementation should be directed. Approximately ten to fifteen women will be interviewed until no new information emerges during the interviews.

#### Outcome measures

The main outcome measures are the barriers and facilitators for adherence to the quality indicators for performing a CS.

#### Data analysis

Using Atlas, the qualitative software package, a qualitative analysis will be performed on the barriers and facilitators that are presented in the interviews among healthcare professionals and patients. The transcribed interview will be marked and coded with barriers and facilitators according to the framework used to structure the interviews: features of the guidelines, professionals, patients, social setting, and organization. These influencing factors will be quantified among all Dutch gynaecologists and midwives by means of questionnaires. The analyses of the questionnaires will be descriptive (*e.g.*, frequencies and means).

### Controlled before-and-after study

#### Design and methods

Based on the results of the current care study and the barrier and facilitator study, one or more target groups for a tailor-made implementation strategy will be identified. Target groups will be selected with focus on women with both a high incidence of the indicator (our hypothesis is that this will include, for example, non-progressing labour and previous CS) and low indicator adherence. A tailor-made implementation strategy will be developed in order to increase adherence to the CS quality indicators. This strategy will be executed and evaluated in a clustered CBA study in 12 hospitals (six intervention, six control hospitals) (see sample size calculation) in terms of effectiveness, experiences, and costs. It is likely that a strategy with different implementation elements is needed because several barriers for implementation of recommendations may exist at different levels. This will probably result in a combined intervention directed at the level of professionals, patients, and the organisation.

#### Study population and setting

The implementation strategy will be executed and evaluated in 12 hospitals (see sample size calculation) that also participated in the current care study: six intervention hospitals in which the newly developed strategy will be applied; and six control hospitals in which care as usual will be offered. In order to select these 12 hospitals, all 20 hospitals of the current care study will be categorized into university, non-university teaching, and non-university non-teaching hospitals (three categories). Within these three categories, possible hospital pairs will be made based on pre-intervention adherence to quality indicators and CS rates, as measured in the current care study. To get a sample representative for the Dutch setting, in total two university hospitals, six non-university teaching hospitals and four non-university, non-teaching hospitals will be asked to participate. Subsequently, the participating hospitals have to be assigned to the intervention and control group. This will be done per stratum and based on geographic region. The evaluation will include an effect, process, and cost analysis. Just as in de current care study, the effects will be measured both at medical outcome level (*i.e.,* CS rates and complication rates) and on guideline adherence level. Satisfaction with and applicability of the tailor-made implementation strategy for both patients and healthcare professionals will be measured in a process evaluation. Information regarding the process will be gathered in a qualitative study in the hospitals in which the implementation strategy was applied. Individual interviews will take place among the involved healthcare professionals and patients to gather data about experiences with the changed care. During the interview, they also will be asked about which elements of the tested strategy they specifically used to implement the evidence-based guidelines; how satisfied they are with the different elements; and their opinion about the feasibility of the different elements.

Furthermore, a cost analyses of the tested implementation strategy will take place with respect to three aspects: 1) the rate at which the guideline recommendations are already applied; 2) the costs of the implementation strategy (taking into account the development of the strategy, training of healthcare professionals, and possible extra costs regarding both time and medical costs) and 3) the effectiveness of the implementation strategy. In order to measure the effectiveness of the implementation strategy both the effects on medical outcome (*i.e.*, CS rates, complication rates) and adherence to CS quality indicators will be measured. The cost-effectiveness of the implementation strategy will be expressed as the incremental costs per extra patient treated according to the CS quality indicators, compared to the ‘do-nothing’ strategy.

#### Outcome measures

The primary outcome is effectiveness of the implementation strategy, which is defined as the observed increase in adherence to the developed quality indicators with regard to the chosen target group (for example non-progressing labour or previous CS) between the intervention and control hospitals and the actual CS rates in both groups. Secondary outcome measures are experiences and satisfaction of healthcare providers and patients with the implementation strategy as well as applicability and costs.

#### Sample size considerations

For a sample size calculation, the target group and the adherence to the quality indicators regarding this target group are necessary. These data will be available after performing the current care as well as the barrier study. Based on these data, the sample size can be calculated. In this calculation, we will take into account clustering of patients within professionals and hospitals. We expect most of the clustering at professional level and presume an inclusion of a number of professionals per hospital and a number of patients per professional. For pragmatic reasons, we will include at most 12 out of the 20 hospitals of the current care measurement. This sample of hospitals has to be representative for the Dutch setting, *i.e.*, a total of two university hospitals, six non-university teaching hospitals, and four non-university, non-teaching hospitals.

#### Data analysis

To assess the effectiveness of the implementation strategy, the proportion of patients that are treated in accordance with the guidelines before and after implementation of the guidelines in both the intervention and control hospitals will be measured. Medical outcome measures will include CS rates and maternal as well as neonatal complications related to vaginal delivery or CS. Multilevel multivariate analysis will be carried out to assess the independent effect of the implementation strategy on adherence to the CS quality indicators and medical outcome measures. A qualitative descriptive analysis will be done in order to evaluate the process. Furthermore, by means of a questionnaire, the experiences of healthcare providers and patient satisfaction with the implementation strategy will be evaluated, and the outcomes will be descriptive.

The costs analysis will be performed from a healthcare perspective. The costs of the implementation process will be calculated on the basis of the time and materials invested based on activity-based costing (ABC) approach, focusing on activities performed with costs accumulated at the activity level(s) of the healthcare implementation processes. The costs of implementation of the guidelines and consolidation consist of personnel and material costs. The input of resources will be assessed by collecting volumes of consumed resources, and multiplying these by the price of each resource unit. For collecting information on the input of the resources, registration forms will be completed by the people involved in the implementation and consolidation process. The prices of each resource unit will be based on standard costs [22], market prices, or self-determined costs. The medical costs used in de cost analysis will include CS rates and maternal and neonatal complications related to vaginal delivery or CS. The costs of implementation and costs of the changed medical care will be weighed against the proportion of patients that are treated according to the CS guideline, after implementation. The cost-effectiveness of implementation will be expressed as the incremental costs per extra patient treated according to the CS guideline, compared to the ‘do-nothing’ strategy (*i.e.*, no implementation, for which data before implementation will be used).

#### Ethical considerations

The Medical Ethical Committee (CMO) of Maastricht (azM/UM) approved this study protocol and declared that no ethical approval was necessary (MEC 09-4-047).

## Discussion

The CS rate in The Netherlands is comparatively low compared to other countries, but it is increasing especially in the group of healthy women with a singleton pregnancy in vertex position at term. One would expect this to coincide with improved outcomes for mothers and children, which is, however, not the case [5]. Although many suggestions considering the reason for this rise have been made, the answer is not yet clear. We hypothesize that incomplete guideline adherence is a possible cause for the current increase in CS rate. In this study we will determine current Dutch care regarding CS using quality indicators. We will use national as well as international guidelines to select the recommendations, resulting in at least a set of internationally accepted indicators. Because we will also report the current CS rates by classification into the internationally accepted Robson Criteria, international comparison of incidences of CS rates in different subgroups will be possible. In that way, specific indicators and incidences of those indicators might be applicable elsewhere. Furthermore, we will focus on factors that influence guideline implementation, and thus optimal care in the barrier and facilitator analysis.

Although earlier reviews claimed that multifaceted strategies (combinations of many different interventions) are often effective, Grimshaw found that a higher number of intervention components was not related to higher effectiveness [23]. It seems plausible that combined interventions are only more effective than single interventions, if these are addressed at the specific barriers to change. This is also the conclusion of Chaillet *et al*.: in the obstetric setting in general and the CS setting in particular, prospective identification of efficient strategies and barriers to change is necessary to achieve a better adaptation of intervention and to improve clinical practice guideline implementation [24].

This study will hopefully result in one or more target groups with high incidence and low guideline adherence and the evaluation of an implementation strategy to improve care. For example, non-progressing labour is known to be one of the major reasons to perform a CS. Should guideline adherence in these women be low, an intervention based both on informing women and reminders on optimal care for caregivers could be an option. Another possible target group might be women with a previous CS. Although in general, the VBAC rate was previously reported higher in the Netherlands than in some other countries, recent data are lacking. Improvement of care for these women could consist of a decision aid to improve counselling. Both types of interventions are also possibly effective outside the Netherlands. The ultimate aim of our study is to implement the national and international evidence-based guidelines on CS in all Dutch hospitals in order to reduce the incidence of CS and improve the outcome for mother and child. Furthermore, this study provides a framework for future studies to enable improvement of guideline adherence and reduction of the CS rate.

## Abbreviations

ACOG: American College of Obstetrics and Gynecology; CS: Caesarean section; NGC: National Guideline Clearinghouse; NICE: National Institute of Clinical Evidence; NVOG: Dutch Society of Obstetrics and Gynaecology; RCOG: Royal College of Obstetricians and Gynaecologists; SOGC: Society of Obstetricians and Gynaecologists of Canada.

## Competing interests

The authors declare that they have no competing interests.

## Authors’ contributions

SM, HS and RH conceived of the study and took part in the study design. They were also responsible for drafting the manuscript. All authors are members of the SIMPLE study group and participated in the design of the study. All authors read, edited earlier versions of the manuscript and approved the final manuscript.
